# Tailoring cell sheets for biomedical applications

**DOI:** 10.1002/SMMD.20230038

**Published:** 2024-02-18

**Authors:** Weiwei Chen, Min Nie, Jingjing Gan, Nan Xia, Dandan Wang, Lingyun Sun

**Affiliations:** ^1^ Department of Rheumatology and Immunology Nanjing Drum Tower Hospital Medical School Nanjing University Nanjing China; ^2^ Department of Rheumatology and Immunology The First Affiliated Hospital of Anhui Medical University Hefei China

**Keywords:** biomedical applications, cell sheet technology, regenerative medicine, tissue engineering

## Abstract

Cell sheet technology has emerged as a novel scaffold‐free approach for cell‐based therapies in regenerative medicine. Techniques for harvesting cell sheets are essential to preserve the integrity of living cell sheets. This review provides an overview of fundamental technologies to fabricate cell sheets and recent advances in cell sheet‐based tissue engineering. In addition to the commonly used temperature‐responsive systems, we introduce alternative approaches, such as ROS‐induced, magnetic‐controlled, and light‐induced cell sheet technologies. Moreover, we discuss the modification of the cell sheet to improve its function, including stacking, genetic modification, and vascularization. With the significant advances in cell sheet technology, cell sheets have been widely applied in various tissues and organs, including but not limited to the lung, cornea, cartilage, periodontium, heart, and liver. This review further describes both the preclinical and clinical applications of cell sheets. We believe that the progress in cell sheet technology would further propel its biomedical applications.

## Graphical abstract

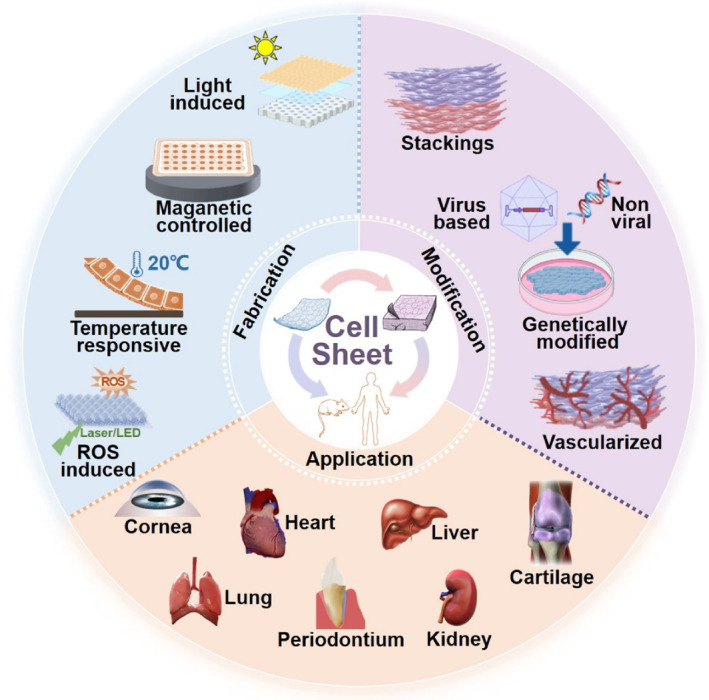

**Key points**

1. The basis of cell sheet engineering is cell sheet harvest technology. The cell sheet can be detached from the special culture surfaces using different stimuli, including temperature variation, magnetic forces, chemical alterations or exposure to light.
2. Stacking, vascularization, and genetic modification enable the fabrication of the cells sheets with complex structures and enhanced functionality.
3. Cell sheet engineering has been a selection of advantages in the field of regenerative medicine. The application of cell sheets in the regeneration of tissues such as the retina and periodontium has entered clinical trails.
4. With the collaboration of various disciplines, exploring new surface materials and more applicable cell resources is essential to develop faster and cost‐effective methods for cell sheet preparation.

## 1 INTRODUCTION

With the development of technology, tissue engineering has displayed considerable promise in the regenerative medicine field.^1^, ^2^, ^3^, ^4^, ^5^ Conventional 3D cell‐seeding scaffolds could not precisely mimic the internal structures and often caused inflammatory responses and fibrosis during their degradation.^6^, ^7^, ^8^ With the rapid progress in tissue engineering, cell sheet technology has been developed as a scaffold‐free method to address these challenges.^4^, ^9^, ^10^, ^11^ The cell sheet can preserve cellular intrinsic physiological functions including intact extracellular matrices (ECM) and cell‐cell junctions by harvesting cells without the use of enzymes. By shaping cells into a sheet, it is easy to transplant to the target site.^12^, ^13^ Consequently, the development of cell sheet engineering is attracting increasing attention.

Cell detachment strategies are essential for cell sheet engineering. Temperature‐responsive systems are most widely used for harvesting cell sheets.^14^, ^15^, ^16^ The surface of culture dishes exhibited a temperature‐sensitive transition between hydrophobic and hydrophilic properties, facilitating cell adhesion or detachment. However, the cell sheet fabrication using a temperature‐responsive system is still costly and time‐consuming.^17^ Other economical cell sheet detachment techniques have also been developed, including magnetic‐controlled,^18^, ^19^ light‐induced,^20^, ^21^ and ROS‐responsive strategies.^22^ The introduction of these novel surface materials facilitates the preparation and harvest of cell sheets, potentially reducing the fabrication time. The construct thickness is increased through stacking single‐layer to multi‐layered cell sheets, subsequently avoiding break and affording more control over cellular interactions. Nevertheless, due to the lack of oxygen or nutrients, the necrosis can occur inside the multilayered cell sheet, resulting in decreased cell viability.^23^ Therefore, vascularized‐layered cell sheets with capillaries carrying oxygen and nutrients have been introduced.^24^, ^25^ Bioprinting is an emerging technology that enables the fabrication of patterned culture surfaces and vessels tailored to the demands of intricate structures for cell‐sheet engineering.^26^, ^27^, ^28^ Furthermore, genetic modification of cells can improve the therapeutic effects of cell sheets.^10^

**FIGURE 1:**
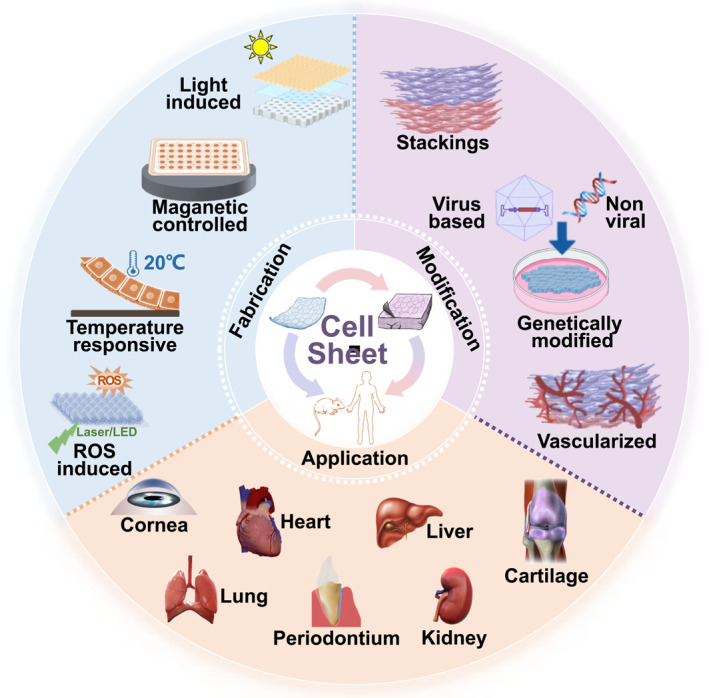
Fabrication and modification of cell sheets and their application in tissue regeneration.

Cell sheet engineering, a promising strategy in the field of regenerative medicine, has been extensively applied in various types of tissue repair and regeneration. The preserved cell‐to‐cell junctions and structures in cell sheets closely mimic that of natural tissues, contributing to the maintenance of cell function.^29^ Moreover, cell sheets have the capability to be directly transplanted into the desired tissue or employed in the construction of 3D structures.^30^, ^31^ Importantly, it avoids the immunogenic reaction caused by scaffold degradation, ultimately enhancing the regenerative capacity. In this review, we first introduce fundamental techniques in the preparation and harvesting of cell sheets. Then, we focus on the modification of the cell sheet to improve its function, such as stacking, genetic modification, and vascularization. Furthermore, we introduce the therapeutic application of cell sheets in different organs or tissues (Figure 1). Finally, we present a summary on the limitations of the current technologies and remaining challenges in cell sheet engineering and propose an outlook on new materials for future applications. Although cell sheet engineering exerts great therapeutic potential, improvements are still needed for widespread clinical translation.

## 2 TECHNOLOGIES FOR CELL SHEET FABRICATION

### 2.1 Temperature‐responsive cell sheet technology

**FIGURE 2:**
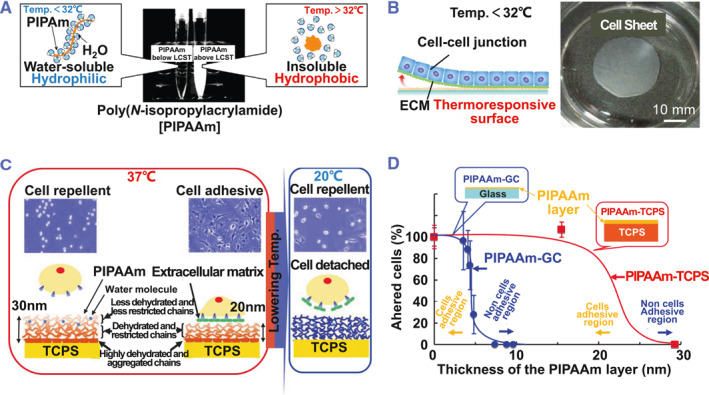
Temperature‐responsive cell sheet technology. (A) Temperature‐dependent alteration of hydrophilicity/hydrophobicity of PIPAAmin aqueous solution. (B) Thermally triggered a detachment of adhering cells from a thermoresponsive culture substrate by lowering the culture temperature. Reproduced with permission.^4^ Copyright 2019, Elsevier. (C) Schematic diagram of molecular motion of plate surface contains covalently‐bound poly N‐isopropyl acrylamide (PIPAAm) chains and cell adhesion/detachment behaviors on the PIPAAm‐grafted surfaces with varying graft thickness. (D) The relationship between the density of spread cells and the thickness of the modified PIPAAm layer. Reproduced with permission.^35^ Copyright 2015, Elsevier.

Temperature‐responsive culture plates are the mainly widely used method for producing cell sheets.^32^ The culture plate surface contains covalently‐bound poly N‐isopropyl acrylamide (PIPAAm), a polymer sensitive to temperature changes.^33^, ^34^ PIPPAm exhibits a critical solution temperature below 32°C (Figure 2A).^4^ The surface of the culture plate transitions from hydrophobic to hydrophilic at temperatures lower than 32°C, allowing cell adhesion‐detachment transition. The temperature‐triggered detachment allows the collection of intact cell sheets with the ECM at the bottom (Figure 2B), which can directly adhere to the target organ.

The thickness of the PIPAAm is a key parameter in determining the hydrophobic or hydrophilic state.^36^ After electron‐beam (EB) irradiation to the monomer solution, PIPAAm was covalently grafted on the culture surface. Through adjusting the EB irradiation conditions or monomer concentration, precise grafting at a nano‐scale can be realized to achieve the optimal thickness of PIPAAm layer, providing the appropriate condition for cell adhesion.^17^, ^37^ Previous studies have reported that the PIPAAm layer with a thickness of about 20 nm is suitable for both cell adhesion and detachment.^35^, ^37^ When the PIPAAm layer reaches a thickness of 30 nm, cells fail to adhere to the PIPAAm‐modified tissue culture polystyrene (TCPS) surface even at 37°C (Figure 2C). Phenomena that the thickness of grafted PIPAAm layers dominates cell behavior have also been observed in PIPAAm‐grafted glass surfaces, in which 4.8 nm is the optimal grafted polymer thickness (Figure 2D).^35^, ^38^

### 2.2 Light‐induced cell sheet technology

**FIGURE 3:**
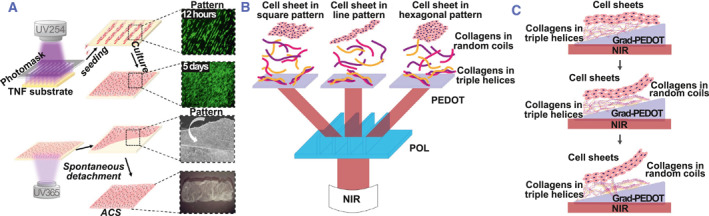
Light‐induced cell sheet technology. (A) Cell sheet patterning induced by ultraviolet 254 (UV254) on titanium oxide (TiO2) nanodots film (TNF). Anisotropic cell sheet (ACS) detachment induced by UV365 on TNF. Reproduced with permission.^21^ Copyright 2017, American Chemical Society. (B) Schematic diagram for the harvest of multiple cell sheets using near‐infrared (NIR) light. (C) Schematic diagram of the precise directional cell sheet detachment from the gradient photothermal surface. Reproduced with permission.^20^ Copyright 2021, Elsevier.

Energy transfer in electrons on the surface also contributes to the cell sheet detachment. Typically, the ECM and the titanium oxide (TiO2) nanodot surface carry a positive charge and exhibit hydrophobic properties, allowing cell adhesion. However, following exposure to UV irradiation, the accumulation of electrons on the surface leads to a negative charge, forming a repulsive force that dislodges the cell sheet from the surface.^39^, ^40^ TiO2 films were prepatterned on the surface and cell sheet adhesion‐detachment transition can be obtained with a two‐step illumination from ultraviolet 254 (UV254) to UV365. HFF‐1 cells were cultured with regular orientation on the film under UV254 irradiation, while detached as a monolayer cell sheet from the surface after 20 min of UV365 irradiation (Figure 3A).^21^

Near‐infrared (NIR) light was also reported to trigger the cell sheet release.^41^, ^42^ Upon NIR‐II laser irradiation, the poly (3,4‐ethylenedioxythiophene) (PEDOT) film exhibited photothermal conversion and enhanced temperature, resulting in the dissociation of the adsorbed collagens from the surface. Subsequently, the cell sheet detached, maintaining cell viability and cell‐cell interactions. The creation of photothermal pattern in the NIR range using a patterned optical lens (POL), contributing to the formation of photothermal pattern (PTP) on the PEDOT film. As a result, cell sheets can be harvested after NIR exposure in different patterns, including squares, lines, or hexagonal patterns (Figure 3B).^43^ Large‐scale production of cell sheets can be accomplished with NIR light‐induced stimulation on distinct culture dishes with different distances between the PEDOT and POL surfaces. hADSC was seeded with fibronectin on the PEDOT film, supporting the formation of a confluent cell sheet that could be detached from the surface after NIR illumination. The collagen dissociation speed depends on the temperature on the surface controlled by the thickness of the PEDOT substrate.^44^ Thus, the cell sheet detached more rapidly from the thicker PEDOT film (Figure 3C), implying that cell sheet detachment can be manipulated spatiotemporally.

### 2.3 Magnetic‐controlled cell sheet technology

**FIGURE 4:**
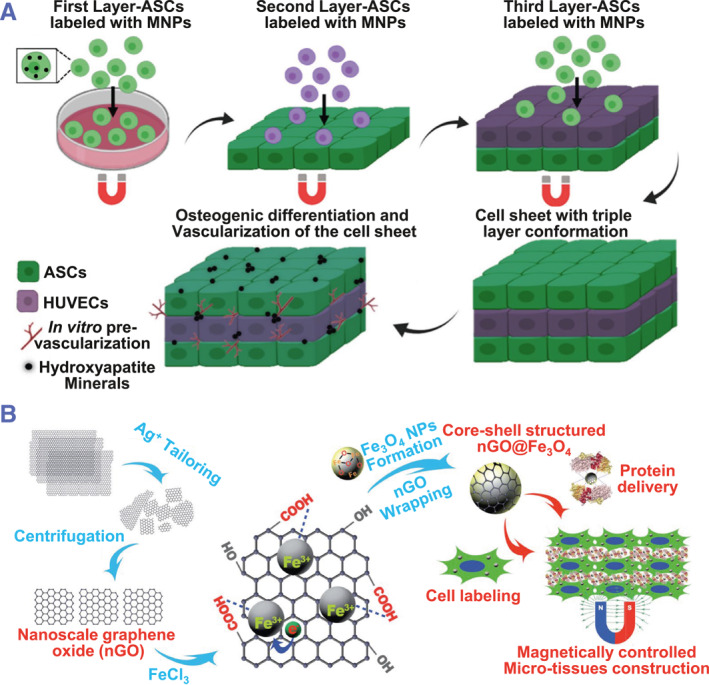
Magnetic‐controlled cell sheet technology. (A) Schematic illustration of the fabrication of the 3D vascularized heterotypic cell sheet using magnetic nanoparticles (MNPs). ASCs, adipose‐derived stromal cells. Reproduced with permission.^25^ Copyright 2020, Elsevier. (B) Schematic illustration of the preparation of the nGO@Fe3O4 MNPs and their application in magnetically controlled growth‐factor‐immobilized multilayer cell‐sheet fabrication. Reproduced with permission.^46^ Copyright 2017, John Wiley and Sons.

To fabricate magnetically controlled cell sheets, magnet‐labeled cells were initially seeded onto the culture surface. Subsequently, the cell sheets can be detached by removing the magnet that is placed under the surface. Detrimental effects of magnetic nanoparticles (MNPs) have not been observed in terms of cell viability and cell adhesion. The magnetically‐controlled cell sheets possess good biocompatibility and exert the potential in regenerative therapy.^45^, ^46^ Since the cell adhesion is controlled by the external magnetic force, layered cell sheets can be formed by regulating the seeded cell number rather than stacking the cell sheets. Thus, magnetic‐controlled cell sheet technology has an advantage in controlling the thickness of the cell sheets.^47^, ^48^ By using magnet‐based techniques, several cell sheets have been fabricated, including stromal cells and endothelial cells (Figure 4A).^25^

Except for the traditional MNPs, graphene oxide (GO)‐coated Fe3O4 MNPs (nGO@Fe3O4) with protein‐delivery function has also been developed. Graphene oxide has been widely used in medical and biological research, exhibiting excellent biocompatibility and special properties in delivering proteins. The nGO@Fe3O4 cell sheet was prepared by wrapping Fe3O4 MNPs in GO sheets (Figure 4B).^46^ The retaining magnetic property of nGO@Fe3O4 enables the control of the thickness of the cell sheets, while the GO allows the delivery of small molecules and cytokines to promote tissue repair and regeneration. Although magnetic‐controlled cell sheet technology is effective and low‐cost, cellular aggregate clumps are easily formed instead of monolayer cell sheets. Furthermore, the unknown effects of the modified cell sheets are undesirable in clinical settings.

### 2.4 Reactive oxygen species‐induced cell sheet technology

**FIGURE 5:**
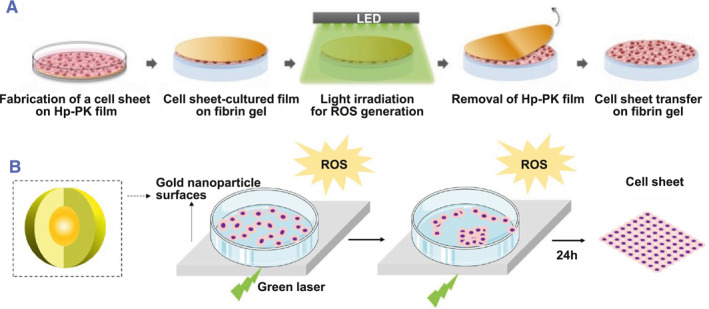
Reactive oxygen species (ROS)‐induced cell sheet technology. (A) Schematic diagram of the ROS‐induced cell sheet detachment and transfer procedure on Hp‐PK films. Reproduced with permission.^13^ Copyright 2019, Elsevier. (B) Process of cell sheet detachment through ROS generation on AuNP surface. Reproduced with permission.^53^ Copyright 2022, Elsevier.

Excess Reactive oxygen species (ROS) induces the reduction of adhesion proteins and results in cell detachment.^13^, ^22^, ^49^ Thus, ROS‐responsive methods also serve as strategies in preparing the cell sheet. Due to the good mechanical performance of the ematoporphyrin‐incorporated polyketone film (Hp‐PK film), cells cultured on the surface are easy to manipulate.^47^, ^50^, ^51^, ^52^ In addition, cell viability was not affected when cultured on Hp‐PK film. Based on the Hp‐PK film, a ROS‐responsive strategy to detach the cell sheet has been established. Under the irradiation of green LED light, exogenous ROS was produced and subsequently triggered the cell sheet detachment from the Hp‐PK film (Figure 5A), which was gently removed finally.^13^ The production of ROS can be controlled by adjusting the irradiation length and light density.

AuNPs are commonly used photosensitizers with triangular prism structures.^54^, ^55^ Upon green laser irradiation, AuNPs are excited after the absorption of energy from the light. Electrons or energy are transferred between oxygen and excited AuNPs, leading to the generation of ROS. The ROS, in turn, disrupts the membrane, separating the cell sheet from the culture surface within 24 h (Figure 5B).^53^ This technique offers the advantage of simplified transplantation. The amount and production rate of ROS, which depend on the laser power, the pattern or concentration of AuNPs, and irradiation duration, are key factors in ROS‐induced cell detachment. Thus, optimizing these conditions can better control the harvest of cell sheets.

Attachment and detachment of cell sheets on the PIPAAm‐graft surface depend on the thickness and density of the grafted polymer chains, which is technically challenging and requires special equipment. Although TCPS culture surfaces grafted with PIPAAm are commercially accessible and simplify the preparation of cell sheets, these culture surfaces are costly.^56^, ^57^ Alternative techniques for harvesting cell sheets, such as light‐induced, magnetic‐controlled, and ROS‐induced methods, have been developed. Nevertheless, despite the successful harvesting of cell sheets, these methods have certain drawbacks. Residual materials may persist with detached cell sheets in magnetic‐controlled methods,^25^, ^58^ while the light‐induced and ROS‐induced method has the potential to harm cells.^21^, ^22^ The advantages and disadvantages of responsive systems are listed in Table 1.

**TABLE 1:** Advantages and disadvantages of the different technologies for cell sheet fabrication.

Responsive systems	Trigger of detachment	Advantages	Disadvantages	Refs
PIPAAm‐grafted surface	Temperature reduction	Non‐invasive harvesting and manipulation, commercially available, intact harvesting, effective detachment	Complicated and time‐consuming grafting method, high cost, specificity of application in certain types of cell sheets, long‐term biocompatibility concerns	56, 57, 59
Light‐induced	Light exposure	Non‐invasive control, low running cost, rapid detachment	Potential photo‐toxicity on cell viability, equipment complexity, challenges in 3D applications	21, 60, 61
Magnetic‐controlled	External magnet	Suitable for construction of multilayer cell sheets, economical and time‐saving	Potential cytotoxicity of magnetic residues, cellular response variation	25, 58
ROS‐induced	ROS inducers	Non‐invasive manipulation, stimulus responsiveness, integration with biological systems	Influence on cell behavior, standardization challenges, cytotoxicity of high levels of ROS	22, 49

## 3 FUNCTION IMPROVEMENT OF CELL SHEET

### 3.1 Cell sheet stackings

**FIGURE 6:**
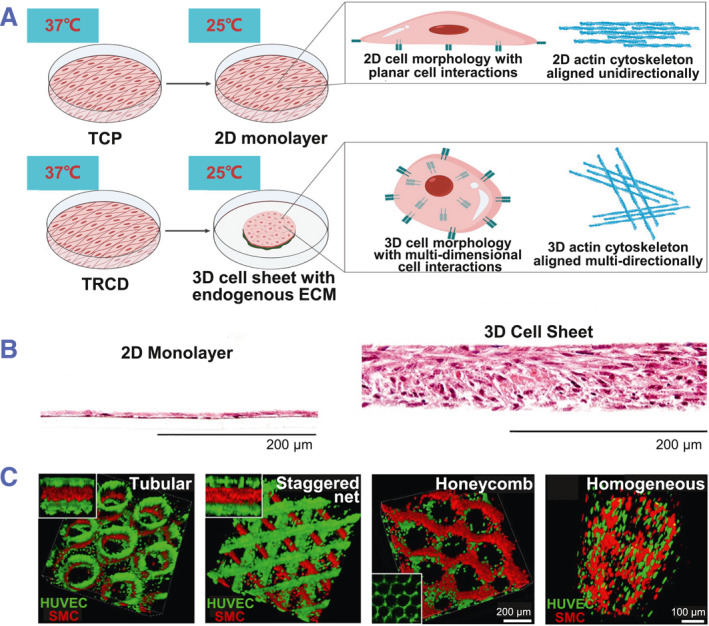
Cell sheet stackings. (A) Spontaneous cell sheet contraction contributes to a 3D tissue‐like structure. (B) Cross‐sectional visualization of both 2D monolayer and 3D cell sheet tissue structure with H&E staining. Reproduced under terms of the CC‐BY license.^65^ Copyright 2021, The Authors, published by Springer Nature. (C) Assembly of multi‐layer cellular construct. Reproduced with permission.^66^ Copyright 2022, IOP Publishing.

The stacking technology is used to fabricate multi‐layered cell sheets or 3D tissues for by layering sheets.^62^, ^63^, ^64^ To prepare the stacking manipulator, gelatin gel was put on thermos‐responsive surfaces. Then, the cell sheets adhering to the manipulator can be stacked onto homotypic or heterotypic layers. The cell layers were incubated at 20°C for 20 min to help attachment and maintain the stability. Finally, the gelatin was dissolved in the heated medium. By structural and morphological transitions, mesenchymal stem cell monolayer contract spontaneously to produce 3D constructs in the absence of a stacking manipulator (Figure 6A).^65^ During such cell sheet fabrication, cell‐cell communications are enhanced with increased intercellular force. The increased thickness of 3D cell sheets was visualized through hematoxylin and eosin (H&E) staining, confirming the transition from single‐nuclei thick to multi‐nuclei thick structures (Figure 6B).^65^ As presented in the confocal images in Figure 6C, the staggered network 3D constructs are assembled flexibly by organizing different layers with different orientations to meet different needs.^66^

3D constructs made by stacking cell sheets are not only beneficial for tissue repair, but also provide tissue/organ models for in vitro studies to uncover the pathogenesis and screen efficient therapies for diseases.^29^, ^30^ The fabrication of 3D tissues with biomimetic structures is far more complicated than conventional static culture. Multiple bioreactors providing organ‐level physical or mechanical conditions have been developed to fabricate functional 3D tissues. Recently, 3D tissue models have been widely used for drug screening in research institutions and drug development companies.^67^ However, cell necrosis can sometimes happen due to the limited oxygen and nutrients inside multi‐layered cell sheets.^29^

### 3.2 Vascularized cell sheet

3D and multi‐layered cell sheets need capillaries to supply oxygen and nutrients for maintaining their functions and viability.^68^, ^69^ When co‐cultured with cardiac cells, endothelial cells can spontaneously form reticular structures.^70^ Angiogenic cytokines, including vascular endothelial growth factor, are secreted by cells co‐cultured with endothelial cells, regulating blood vessel formation.^71^, ^72^, ^73^ Cell sheets co‐cultured with endothelial cells are highly vascularized to offer sufficient oxygen and nutrients and show high transplantation efficiency.^74^, ^75^ The results indicate the critical role of vascularization in successful transplantation of highly functional multi‐layered and 3D cell sheets.

**FIGURE 7:**
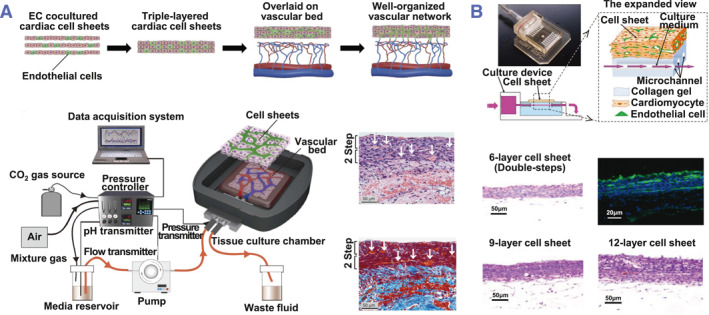
Vascularized cell sheet. (A) Schematic illustration of a bioreactor construct with a vascular bed for perfusion culture medium for layered cell sheets containing endothelial cells. Reproduced with permission.^76^ Copyright 2013, The Authors, published by Springer Nature. (B) Collagen gel with flow channels is used as a vascular bed for vascularized myocardial tissue fabrication. Reproduced under terms of the CC‐BY license.^77^ Copyright 2013, The Authors, published by Springer Nature.

In tissue engineering, bioreactors that replicate in vivo conditions have been employed to create functional 3D tissues. These bioreactors provide perfusion, mechanical stretching or hydro‐pressure. After culturing on a vascular bed with connectable arteries and veins, the multiple‐layered cardiac cell sheets that contain vascular endothelial cells can form a blood vessels. In this bioreactor system, culture media are perfused in the vascular bed.^76^ The connections between the newly formed blood vessels within the cell sheet and the vasculatures in the vascular bed confirmed the successful engineering of functional vasculatures in vitro (Figure 7A). In other settings, collagen hydrogel with channel structures is used to perfuse the culture medium for cardiomyocyte sheet containing endothelial cells culturing.^77^ The vascular network can then form between the cell sheet and the flow channels (Figure 7B). Multi‐step transplantation procedure is applied to fabricate the multi‐layered vascularized myocardial tissue.

### 3.3 Genetically modified cell sheets

By manipulating targeted genes, genetic modification has been introduced to enhance the therapeutic capabilities of cell sheets.^78^, ^79^ The fabrication of the genetically modified cell sheets involves gene transfection, which can be conducted on both the dissociated cells and the assembled cell sheets. Due to the ECM and high cell density, the transfection efficiency of the cell sheets is lower than the dissociated cells. However, the lower cell viability of transfected cells prevents the formation of cell sheets.^78^

Two major approaches, including virus‐based and non‐viral gene transfection, have been applied in the genetically modified cell sheet fabrication. Persistent transgene expression is indispensable for the prominent therapeutic effects of cell sheets. With higher transfection efficiency and prolonged gene expression, virus‐based transfection represents the main approach for gene delivery. Vectors such as adenovirus, retrovirus and lentivirus are widely used in gene transfection of the cell sheets. MSCs secreting angiogenic factors or anti‐inflammatory factors were generated by exploiting adeno‐associated virus‐based vector‐mediated genome editing technology. Cell sheets of genome‐engineered MSCs revealed protective effects against renal ischemia/reperfusion injury in mice.^10^ Human cementum protein 1 gene‐modified rat adipose‐derived mesenchymal stem cells sheets based on lentivirus have a marked ability to enhance periodontal regeneration in osteoporosis rats.^80^ However, safety concerns regarding virus‐based transfection, including immunogenicity, oncogenicity and cytotoxicity, are inevitable.^81^, ^82^ Non‐viral vector systems using liposomes and nanoparticles are emerging alternatives to viral‐based systems, with promising advantages in synthesis, packing and safety.^83^, ^84^ By generating plectin‐deficient cells using the CRISP/Cas9 system delivered by lipofectamine transfection, plectin was proven to be essential for cell tension and cohesion in epithelial sheets.^85^ Although showing great potential in clinical applications, these genetically modified cell sheets remain in the experimental stages. Future efforts to improve the safety of gene modification in cell sheets will help to expand its clinical application.

## 4 PRECLINICAL AND CLINICAL APPLICATIONS OF CELL SHEETS

Cell sheets can be transplanted intactly to the target site while preserving cell‐cell junctions and directly without the intervention of artificial scaffolds or additional treatments.^31^ The preserved ECM enables the cell sheets to adhere tightly to the target tissue without suturing or the use of tissue glue.^86^ With these advantages, the cell sheet‐based tissue engineering has great potential in regenerative medicine.^53^, ^87^, ^88^, ^89^ Large number of preclinical and clinical studies have been carried out to demonstrate the therapeutic benefits of the cell sheets in various tissues or organs.^90^

**FIGURE 8:**
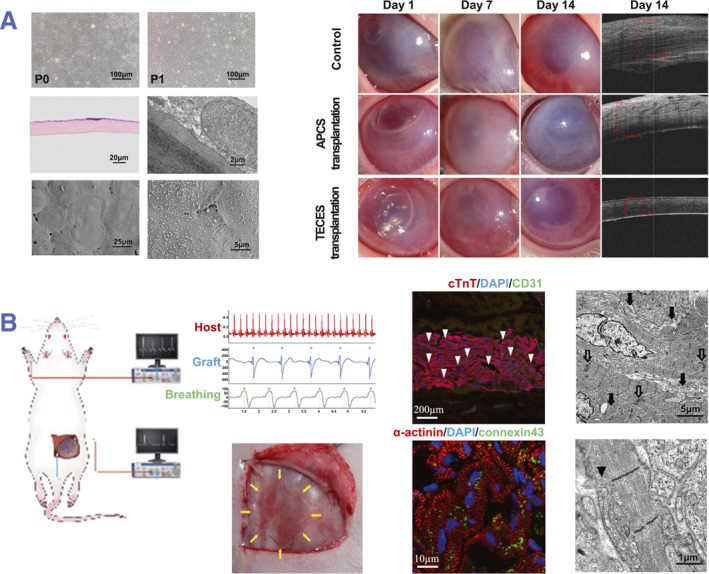
Preclinical applications of cell sheets. (A) Construction of Tissue‐engineered cornea endothelial sheets (TECES) with HCECs and evaluation of the therapeutic effects of TECES transplantation. Reproduced with permission.^117^ Copyright 2022, The Authors, published by American Chemical Society. (B) Fabrication of cardiomyocyte sheets using human induced pluripotent stem (iPS) cells and evaluation of cardiac function in vivo. Reproduced with permission.^118^ Copyright 2017, John Wiley and Sons.

Cell sheet technology has been widely used in a variety of organs, such as the heart,^12^, ^14^, ^91^, ^92^, ^93^ cornea,^94^, ^95^, ^96^ cartilage,^97^, ^98^ periodontium,^99^, ^100^ lung,^101^, ^102^ ear,^103^ liver^104^, ^105^ and kidney^10^, ^106^ (Table 2). Cell sheet transplantation can supplement cells directly and release cytokines in the targeted organ, accounting for the therapeutic efficacy in tissue and organ repair. Sufficient cells are essential for tissue regeneration of damaged organs. Cell sheet technology has proven successful in replenishing cells at affected sites in numerous instances. Tissue‐engineered cornea endothelial sheets have been developed to supply abundant functional endothelial cells to restore damaged cornea.^117^ After transplantation, the Tissue‐engineered cornea endothelial sheets adhered to the posterior stroma, restoring the thickness of donor corneas to normal levels and consequently improving vision (Figure 8A).

**TABLE 2:** Preclinical and clinical applications of cell sheets.

Tissue	Dysfunction	Cell types	Fabrication technologies	Stage of study	Refs
Heart	Ischemic myocardium	Skeletal cells	Temperature‐responsive system	Preclinical	107
	Dilated cardiomyopathy	Autologous myoblast	Temperature‐responsive system	Clinical	108
	Myocardial infarction	Adipose‐derived regenerative cells	Magnetite tissue engineering technology	Preclinical	48
	Myocardial infarction	Mesenchymal stem cells	Temperature‐responsive system	Preclinical	14, 92
Eye	Limbal stem‐cell deficiency	Autologous oral mucosal epithelial cells	Temperature‐responsive system	Clinical	94
	Limbal stem cell deficiency	Adipose mesenchymal stem cells	Temperature‐responsive system	Preclinical	96
Cartilage	Cartilage damage	Chondrocyte	Temperature‐responsive system	Preclinical	109
	Knee osteochondral defects	Chondrocytes	Temperature‐responsive system	Preclinical	110
	Tracheal defects	Chondrocytes	Temperature‐responsive system	Preclinical	111
Periodontal tissue	Periodontitis	Periodontal ligament (PDL)‐derived cells	Temperature‐responsive system	Clinical	112
	Periodontal disease	Periodontal ligament cells and osteoblast‐like cells	Temperature‐responsive system	Preclinical	99
	Periodontal intrabony defects	Adipocyte‐derived dedifferentiated fat cell	Temperature‐responsive system	Preclinical	113
Lung	Pulmonary air leaks	Lung derived cells	Temperature‐responsive system	Preclinical	101
	Thoracoscopic lung resection	Fibroblasts	Temperature‐responsive system	Clinical	114
	Lung injury	Alveolar epithelial cells	Temperature‐responsive system	Preclinical	102
Ear	Otitis media surgery	Middle ear mucosal cell	Temperature‐responsive system	Preclinical	115
	Middle ear cholesteatoma, adhesive otitis media	Nasal mucosal epithelial cell	Temperature‐responsive system	Clinical	103
Liver	Acute liver failure	Human induced pluripotent stem cell‐derived hepatocyte	Temperature‐responsive system	Preclinical	105
	Liver fibrosis	Mesenchymal stem cells	Temperature‐responsive system	Preclinical	104
Kidney	Renal dysfunction	Renal cell	Temperature‐responsive system	Preclinical	116
	Acute kidney injury	Mesenchymal stem cells	Temperature‐responsive system	Preclinical	10
	Ischemia‐reperfusion injury	Mesenchymal stem/stromal cell	Temperature‐responsive system	Preclinical	106

In addition, the paracrine effects of cytokines are crucial for cell sheet regenerative therapies. The cell sheet‐derived cytokines facilitate the self‐repair of the targeted tissues. Cardiomyocyte sheets were developed using cardiomyocytes derived from human induced pluripotent stem cells and were transplanted to improve cardiac dysfunction.^118^ The graft with vascular networks and sarcomere keeps beating for over 6 months independent of the respiratory rhythm or heart rate of the host (Figure 8B). Through the paracrine of the repair factors, the cardiac cell sheets show great prospects in cardiac diseases.^92^

Ebihara et al demonstrated the repair of cartilage by the transplanted autologous cartilage cell sheet‐derived cytokines, and this procedure has also been performed to treat cartilage abrasion caused by osteoarthritis in a clinical study.^109^ Iwata et al showed regeneration the periodontal tissue following autologous periodontal ligament cell sheet transplantation to treat advanced periodontal disease.^112^ Clinical studies of this technique have been conducted. Sekiya et al fabricated the renal cell sheet with adult porcine renal cells. After the transplantation to the damaged kidney, the cell sheet continuously released cytokines, such as active vitamin D3 and erythropoietin, allowing the recovery of renal function.^116^

These findings demonstrate the effectiveness of cell sheet therapy in various diseases, not just by supplementing cells to the targeted organ, but also through the advantageous paracrine effects of the cell sheet. There is a reasonable prospect that the cell sheet therapy will provide more effective therapeutic outcomes compared with conventional treatments. We anticipate further broader clinical applications of cell sheet therapy in various medical fields.

## 5 CONCLUSIONS

Cell sheet technology has shown great promise in the field of regenerative therapy. In this review, we provide a summary of the reported techniques to fabricate cell sheets. A multitude of techniques and concepts have been utilized to fabricate cell sheets, drawing from various technologies and approaches. Moreover, with the progress in cell sheet engineering, more technologies have been used to enhance the functions and improve the therapeutic effects of cell sheets. Based on our review, the users can choose a suitable method or combine multiple methods considering their advantages to fabricate different cell sheets. Finally, we reviewed the applications of cell sheets for various tissue reconstructions. Cell sheets have shown promising potential for restoring tissue functions. We believe that advances in cell sheet technology will always improve the regenerative capacity of the cell sheets. Future research should focus on the action mechanism, improvement and standardization of the fabricating techniques of the cell sheets.

## AUTHOR CONTRIBUTIONS

Weiwei Chen and Nan Xia wrote the manuscript. Min Nie and Jingjing Gan participated in searching for literature and edited the paper. Nan Xia, Dandan Wang, and Lingyun Sun contributed to the study design and conducted a critical review of the manuscript.

## CONFLICT OF INTEREST STATEMENT

The authors declare that there are no competing interests.

## Supporting information

Supplementary Material
