# Supplementary material for: Tailoring cell sheets for biomedical applications
Source: Smart Med. 2024 Feb 18;3(1):e20230038. doi: 10.1002/SMMD.20230038 (PMC11235941; doi:10.1002/SMMD.20230038)

Copyrights for Figure 2a, b


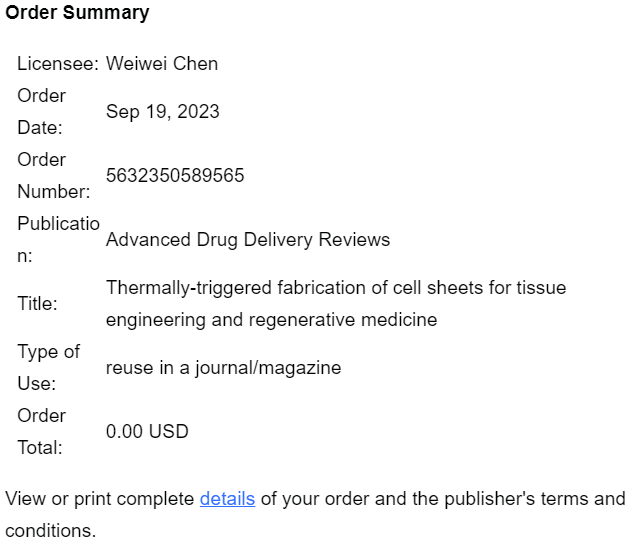


Copyrights for Figure 2c, d


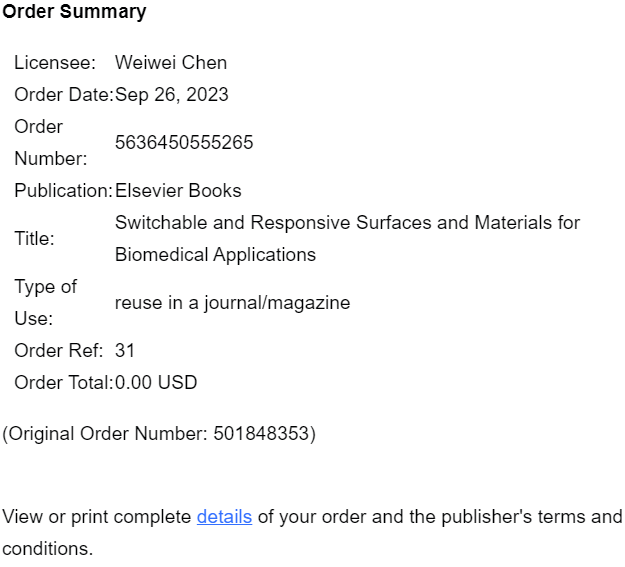


Copyrights for Figure 3a


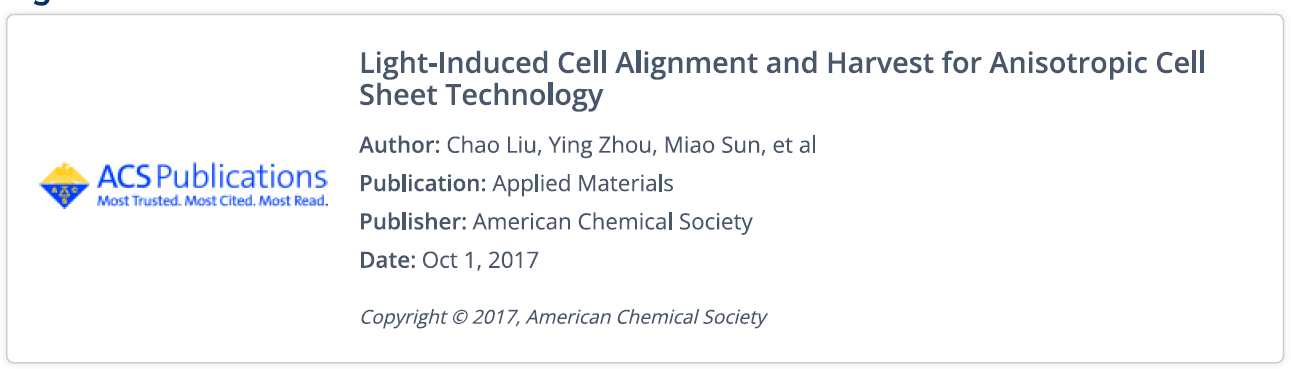


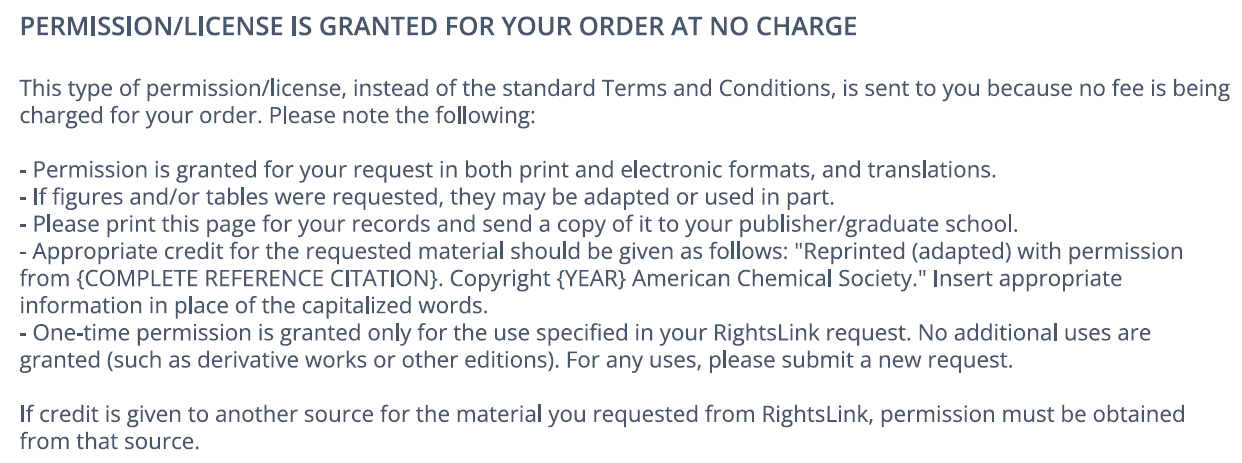


Copyrights for Figure 3b, c


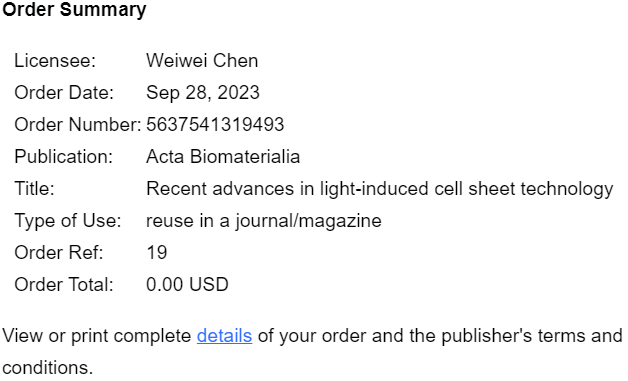


Copyrights for Figure 4a


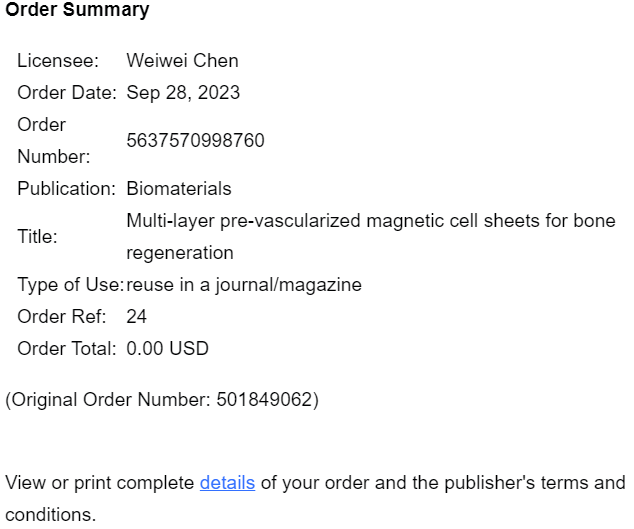


Copyrights for Figure 4b


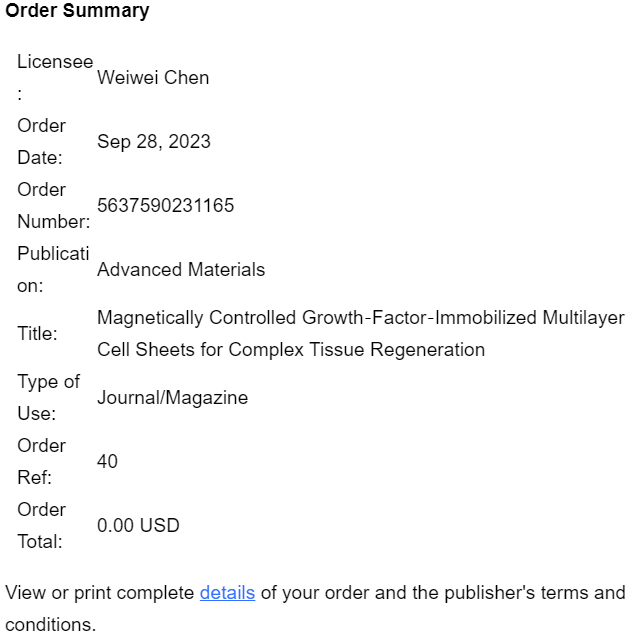


Copyrights for Figure 5a


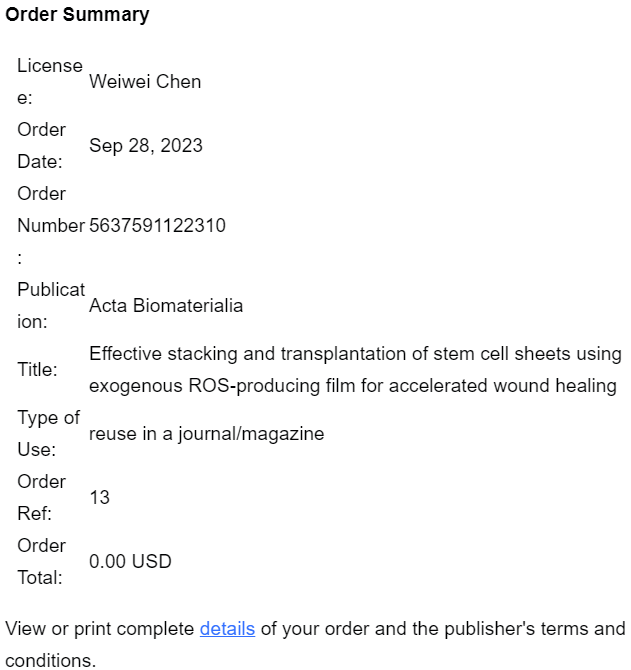


Copyrights for Figure 5b


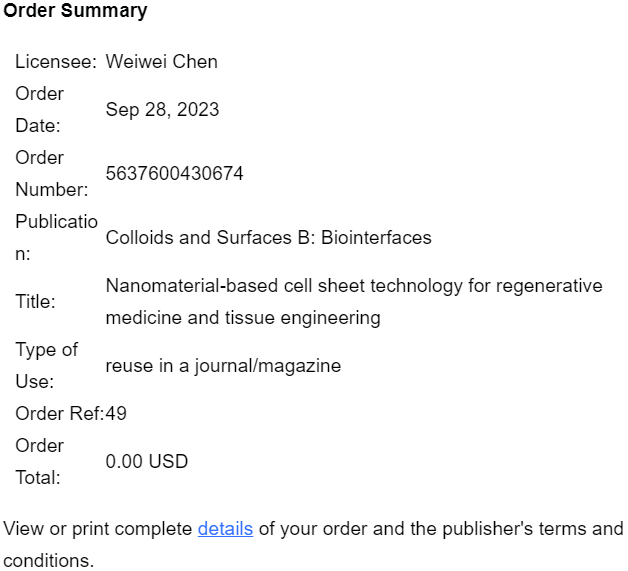


Copyrights for Figure 6 a, b


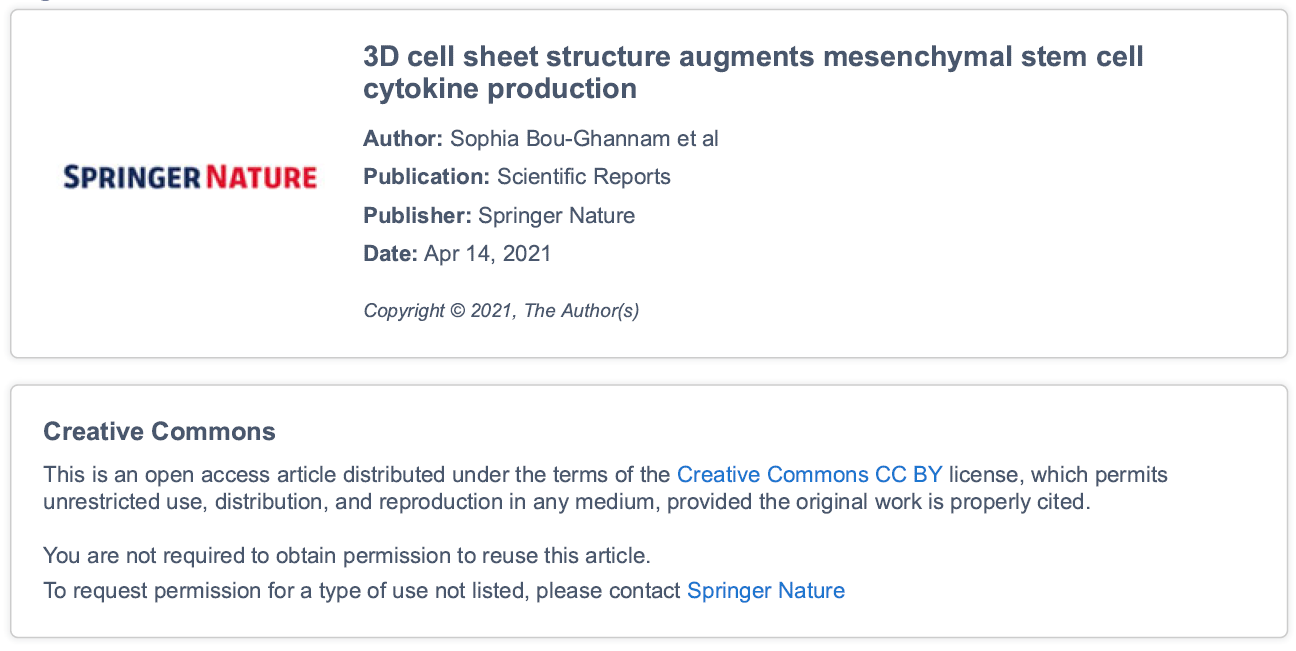


Copyrights for Figure 6c


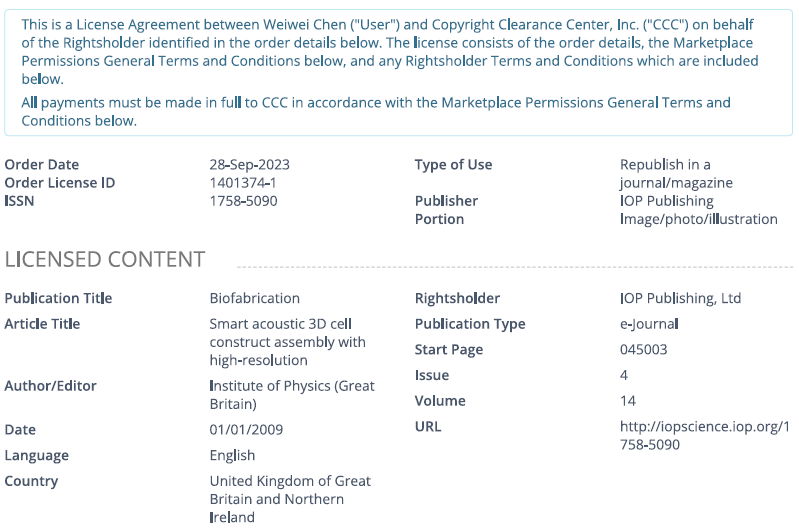


Copyrights for Figure 7a


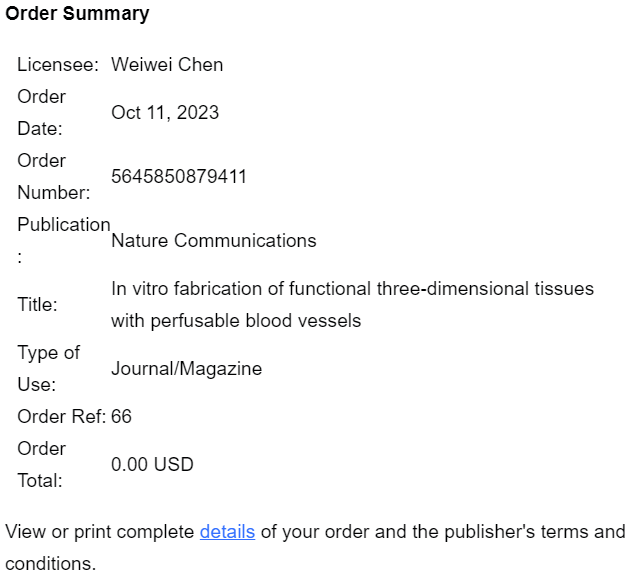


Copyrights for Figure 7b


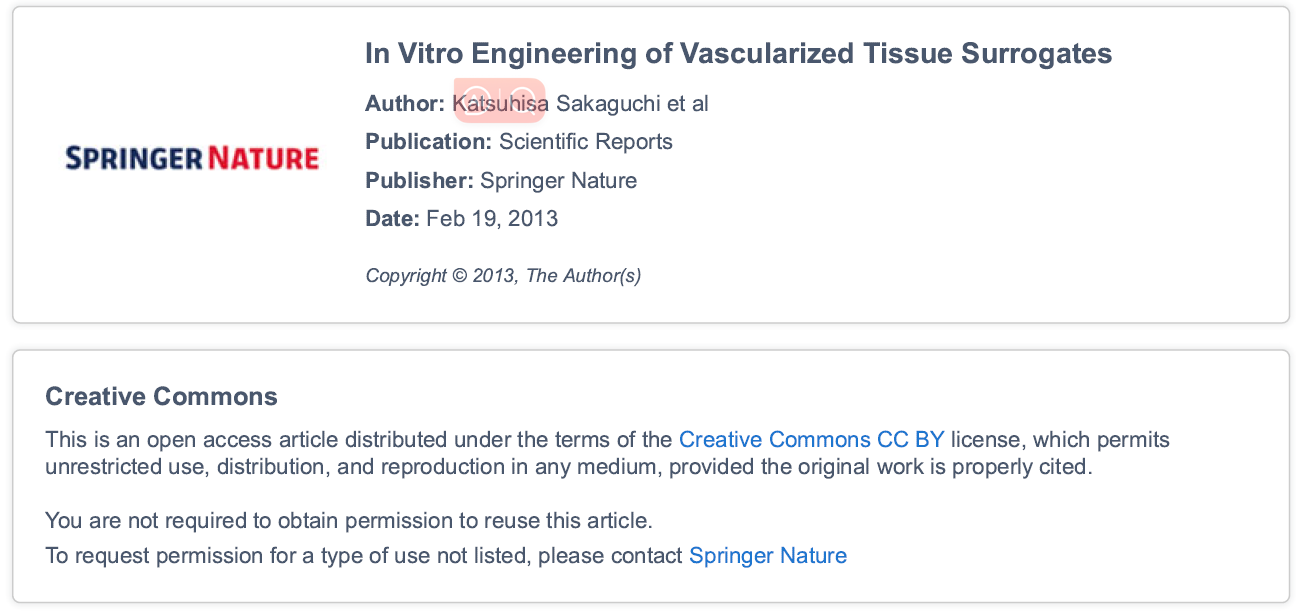


Copyrights for Figure 8a

**Tissue-Engineered Corneal Endothelial Sheets Using Ultrathin Acellular Porcine Corneal Stroma Substrates for Endothelial Keratoplasty**

Yingying Zhang, Zhixin Hu, Jingyu Qu, Huatao Xie, Jun Zhao, Tingjun Fan, Xin Liu*****, and Mingchang Zhang*****

[**Cite this:**](https://pubs.acs.org/action/showCitFormats?doi=10.1021%2Facsbiomaterials.2c00039&href=/doi/10.1021%2Facsbiomaterials.2c00039)*ACS Biomater. Sci. Eng.* 2022, 8, 3, 1301–1311

Publication Date:March 1, 2022

<https://doi.org/10.1021/acsbiomaterials.2c00039>

**Copyright © 2022 The Authors. Published by American Chemical Society. This publication is licensed under**

[**CC-BY-NC-ND 4.0**](https://creativecommons.org/licenses/by-nc-nd/4.0/)**.**

### License Summary*

You are free to share (copy and redistribute) this article in any medium or format within the parameters below:

Creative Commons (CC): This is a Creative Commons license.

Attribution (BY): Credit must be given to the creator.

Non-Commercial (NC): Only non-commercial uses of the work are permitted.

No Derivatives (ND): Derivative works may be created for non-commercial purposes, but sharing is prohibited.

Copyrights for Figure 8b


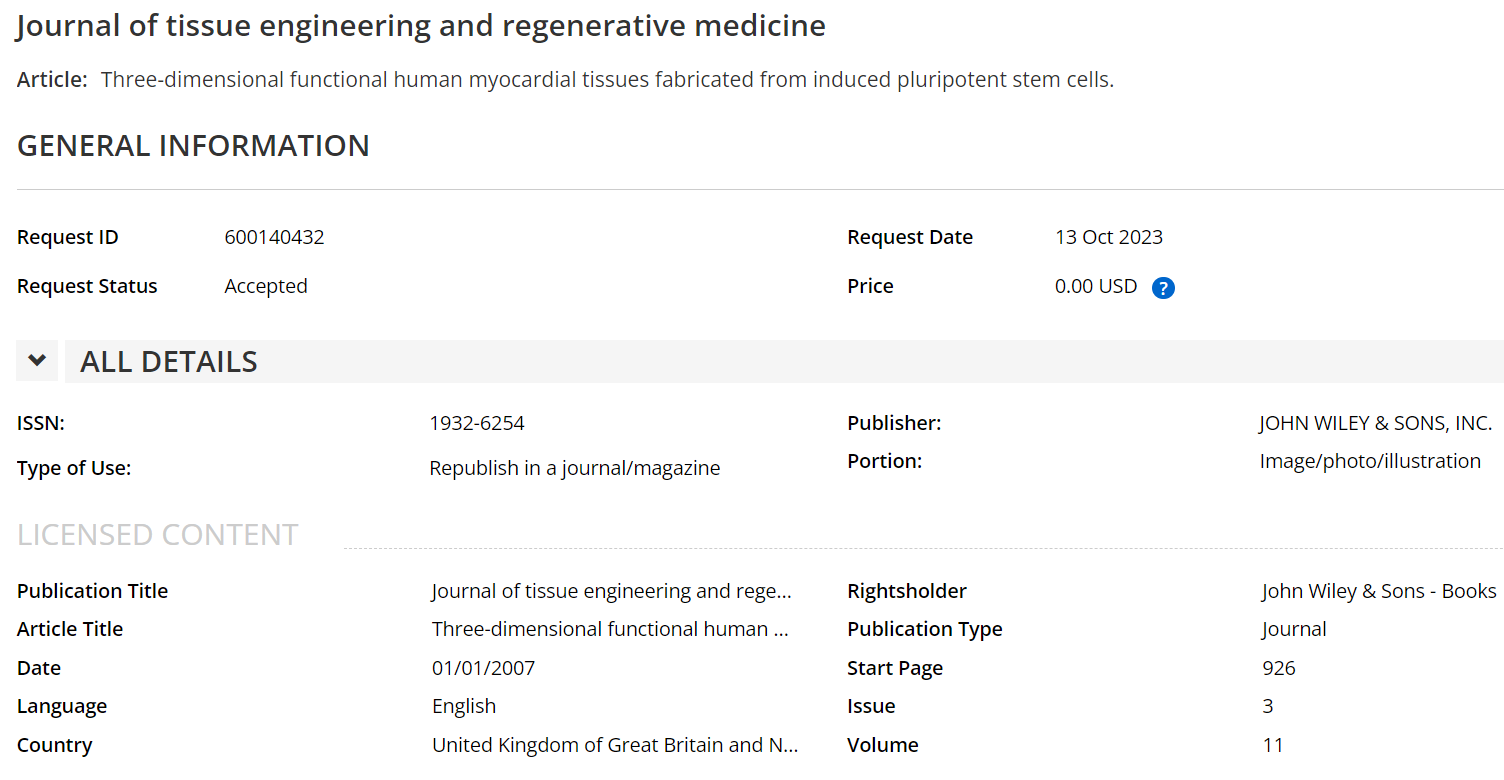


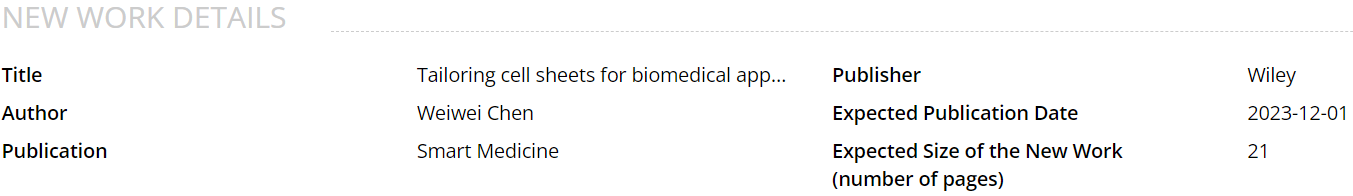

Supplement: Supplementary file 1 — Supplementary Material [file SMMD-3-e20230038-s001.docx]
